# Phase-Controlled NiO Nanoparticles on Reduced Graphene Oxide as Electrocatalysts for Overall Water Splitting

**DOI:** 10.3390/nano11123379

**Published:** 2021-12-13

**Authors:** Seung Geun Jo, Chung-Soo Kim, Sang Jun Kim, Jung Woo Lee

**Affiliations:** 1 Department of Materials Science and Engineering, Pusan National University, Busan 46241, Korea; linkroot1128@pusan.ac.kr; 2 Analysis & Certification Center, Korea Institute of Ceramic Engineering & Technology, Jinju 52851, Korea; 3 Institute of Materials Technology, Pusan National University, Busan 46241, Korea

**Keywords:** dual phase-controlled catalyst, Ni-NiO nanoparticle, overall water splitting, hybrid electrocatalyst, renewable energy

## Abstract

Efficient water electrolysis is one of the key issues in realizing a clean and renewable energy society based on hydrogen fuel. However, several obstacles remain to be solved for electrochemical water splitting catalysts, which are the high cost of noble metals and the high overpotential of alternative catalysts. Herein, we suggest Ni-based alternative catalysts that have comparable performances with precious metal-based catalysts and could be applied to both cathode and anode by precise phase control of the pristine catalyst. A facile microwave-assisted procedure was used for NiO nanoparticles anchored on reduced graphene oxide (NiO NPs/rGO) with uniform size distribution in ~1.8 nm. Subsequently, the Ni-NiO dual phase of the NPs (A-NiO NPs/rGO) could be obtained via tailored partial reduction of the NiO NPs/rGO. Moreover, we demonstrate from systematic HADDF-EDS and XPS analyses that metallic Ni could be formed in a local area of the NiO NP after the reductive annealing procedure. Indeed, the synergistic catalytic performance of the Ni-NiO phase of the A-NiO NPs/rGO promoted hydrogen evolution reaction activity with an overpotential as 201 mV at 10 mA cm^−2^, whereas the NiO NPs/rGO showed 353 mV. Meanwhile, the NiO NPs/rGO exhibited the most excellent oxygen evolution reaction performance among all of the Ni-based catalysts, with an overpotential of 369 mV at 10 mA cm^−2^, indicating that they could be selectively utilized in the overall water splitting. Furthermore, both catalysts retained their activities over 12 h with constant voltage and 1000 cycles under cyclic redox reaction, proving their high durability. Finally, the full cell capability for the overall water electrolysis system was confirmed by observing the generation of hydrogen and oxygen on the surface of the cathode and anode.

## 1. Introduction

Hydrogen fuel is one of the promising alternative energy resources to replace fossil fuels, because of its intrinsic gravimetric energy density (142 MJ kg^−1^) and zero-emission nature [1,2]. Among various types of hydrogen generation procedures, such as steam reforming, partial oxidation of hydrocarbons, coal gasification, and by-product hydrogen generation from industrial processes [3,4,5,6,7], the electrochemical water splitting is the most favorable method as a clean and facile way in that it only produces hydrogen and oxygen from water molecules under external electric bias [8]. However, it still also demands a large amount of energy to generate hydrogen from water due to its high overpotential from the activation energy barrier of the electrode, the resistance of the electrolyte, and contact resistance at the electrode interface [7]. To this end, it is necessary for electrocatalysts to overcome the limitations that would enable the reduction in applied electric potential for enhanced efficiency of water splitting.

Generally, Pt-based electrocatalysts are used for hydrogen evolution reaction (HER) and Ru- or Ir-based materials are applied to oxygen evolution reaction (OER) because of their excellent catalytic activities [9,10,11]. However, they are noble metals, which are very expensive and deficient in the Earth’s crust, thereby hindering commercialized use. Hence, it is imperative to find out Earth-abundant, cost-effective, and highly catalytically active materials such as transition metal-based catalysts [12,13].

Along this direction, many studies have been conducted on electrochemical water splitting using transition metals, which show considerable catalytic activity according to the volcano curve. Among them, Ni is the most promising candidate due to its high electronic conductivity, thermal stability, and alkaline corrosion resistance [14,15]. Moreover, its phase could be tuned by combining with other metals [16,17,18] or non-metals such as nickel sulfide [19,20], nickel selenide [21], and nickel phosphide [22,23] to enhance catalytic performance. In addition, it was reported that the dual-phase of Ni-NiO nanostructures could show synergistic effects in HER [24,25], and also NiO could show suitable activity and durability in OER [9].

On the other hand, graphene is an attractive supporting material of the electrocatalyst to provide a high theoretical specific surface area (2630 m^2^ g^−1^) [26] and intrinsic electron mobility (200,000 cm^2^ V^−1^ s^−1^) [27]. Moreover, reduced graphene oxide (rGO) is one of the graphene materials synthesized by chemically exfoliated procedure, which could be mass productive, preserving the advantages of the graphene. Therefore, rGO is suitable for practical use as supporting material [28,29].

Herein, we report on NiO nanoparticles (NPs) anchored on rGO by facile microwave-assisted synthesis and the control of their interface via reductive annealing. The NPs were anchored on the rGO surface in a uniform size, and they showed high catalytic activity, preventing aggregation during a chemical reaction. Additionally, a partial reduction step processed by post-annealing resulted in co-existing phases of nickel and nickel oxide. We found that annealed NiO NPs showed superior performance in HER, while as-synthesized NPs represented higher activity in OER than other catalysts. This suggests that each catalyst could be adopted selectively in both electrodes. In addition, they had excellent durability in alkaline media, maintaining the redox reaction capability for 1000 cycles at a constant potential over 12 h. Finally, these materials were applied to the overall water electrolysis system and confirmed their availability by the generation of hydrogen and oxygen on the individual electrode surfaces.

## 2. Materials and Methods

### 2.1. Synthesis of NiO NPs/rGO

Nickel oxide nanoparticles/reduced graphene oxide (NiO NPs/rGO) was synthesized using a microwave-assisted method. First, 10 mg of rGO (Angstron Materials, Dayton, OH, USA) and 50 mL of diethylene glycol (DEG, Junsei, Tokyo, Japan, 99%) were mixed in a 50 mL glass vial with ultrasonication for 3 h. Subsequently, 1 mL of 50 mM nickel (II) chloride hexahydrate (NiCl_2_∙6H_2_O, Junsei, 97%) and 1 mL of 0.5 M sodium hydroxide (NaOH, Junsei, 97%) were added into the solution, followed by sonication for 1 h. The solution was transferred to a 250 mL round flask and heated in a microwave oven with 700 W for 2 min. After cooling to room temperature, the solution was transferred to a 50 mL conical tube and centrifuged at 8000 rpm for 50 min. Then, the supernatant liquid was decanted, and the residual catalyst was washed with acetone (Daejung, Siheung, Korea, 99.9%) several times. Finally, the samples were dried in a vacuum oven at 60 °C overnight. As a control sample, nickel oxide nanoparticles/graphene oxide (NiO NPs/GO) were also synthesized by the same process.

### 2.2. Synthesis of A-NiO NPs/rGO

Annealed nickel oxide nanoparticles/reduced graphene oxide (A-NiO NPs/rGO) was fabricated by an additional thermal process. The NiO NPs/rGO was placed in a quartz tube furnace and annealed at 400 °C for 3 h under Ar/H_2_ atmosphere with a flow rate of 100 sccm/80 sccm. For comparison, the NiO NPs/rGO was heated at 300 °C (A-NiO NPs/rGO (300 °C)) and 500 °C (A-NiO NPs/rGO (500 °C)) to investigate the effect of the annealing temperature. It was heated only with Ar atmosphere at the same temperature (400 °C) and flow rate (100 sccm) to investigate the effect of the annealing atmosphere. In addition, the NiO NPs/GO was annealed under the same conditions, producing A-NiO NPs/GO.

### 2.3. Materials Characterization

The surface morphologies and elemental compositions were investigated by field emission transmission electron microscopy (FE-TEM, Talos F200X, Thermo Fisher Scientific, Waltham, MA, USA) and an aberration-corrected TEM (Cs-corrected TEM, Themis Z, FEI company, Hillsboro, OR, USA) equipped with energy dispersive spectroscopy (EDS, Super X, FEI company). The chemical states and bonding structures were analyzed using an X-ray photoelectron spectrometer (XPS, K-Alpha, Thermo Fisher Scientific) with a monochromated Al Kα (1486.6 eV). Inductively coupled plasma optical emission spectrometry (ICP-OES, Optima 8300, Perkin Elmer, Waltham, MA, USA) was used to characterize the amount of Ni from the catalysts.

### 2.4. Electrochemical Measurement

All electrochemical performances were evaluated by using a standard three-electrode cell system connected to an electrochemical workstation (VSP, Biologic, Grenoble, France) with a rotating ring-disk electrode rotator (RRDE-3A, ALS, Tokyo, Japan). All used electrodes and voltammetry cells were made of polymer (polyether ether ketone, polymethyl pentene) to exclude the effect of glass dissolution by alkaline electrolytes during the reaction [30,31]. Pt wire (EC Frontier, Kyoto, Japan) and Hg/HgO (20% KOH filled) were used as a counter and a reference electrode, respectively. In addition, 1 M KOH solution (Junsei, Tokyo, Japan, 85%) was used as an electrolyte. To produce a homogeneous ink, 4 mg of catalyst, 1 mL of ethanol (OCI company, Seoul, Korea, 99.9%), and 80 μL of Nafion solution (5 wt %, Alfa Aesar, Haverhill, MA, USA) were mixed by ultrasonication for 30 min. Then, 15 μL of the catalyst ink was drop-casted and dried on a glassy carbon electrode (GCE, 5 mm diameter, ALS, Tokyo, Japan) with ~0.28 mg cm^−2^ loading amount. Both HER and OER catalytic activities were analyzed by linear sweep voltammetry (LSV) at a scan rate of 5 mV s^−1^ with a potential range of −0.70–0.10 V vs. reversible hydrogen electrode (RHE) for HER and 1.10–1.90 V vs. RHE for OER. Tafel slopes were obtained from the corresponding LSV curves. The charge transfer resistance of the catalysts was measured using electrochemical impedance spectroscopy (EIS) in the frequency range from 100 kHz–0.01 Hz. Moreover, turnover frequency (TOF) was calculated to compare the hydrogen turnover rate of each catalyst and detailed calculation was shown in the electronic supplementary information (ESI). The catalytic durability was evaluated from the cyclic voltammetry (CV) measurement at a scan rate of 50 mV s^−1^ for 1000 cycles, and also the chronoamperometry (CA) under a given potential of −0.30 V vs. RHE (HER) and 1.53 V vs. RHE (OER) for 12 h. The measured potentials were calibrated with a reversible hydrogen electrode (RHE) using the following equation:

E_RHE_ = E_Hg/HgO_ + 0.0591 pH + 0.098 (1)

The electrochemical performances of all catalysts, including commercial 20 wt % Pt/C (Alfa Aesar) and 20 wt % Ir/C (Fuel Cell Store, College Station, TX, USA), were evaluated under the same conditions. The overall water splitting experiment was conducted in a two-electrode cell system. Here, Ni foams (1.5 cm × 1.5 cm) were used as current collectors of the working and the counter electrodes. For the general preparation of current collectors, they were washed with ethanol and then immersed in 3 M HCl for 20 min to remove the NiO surface, followed by additional rinsing with the ethanol. As the next step, the pre-treated Ni foams were dip-coated with ~2.0 mg cm^−2^ loading amount of the catalyst ink and dried overnight in a vacuum oven at 60 °C. The catalytic activity was measured by LSV at a scan rate of 5 mV s^−1^ and a potential range of 0.50 to 2.50 V. Finally, the long-term stability of the catalysts was estimated at a potential of 2.00 V for 12 h.

## 3. Results and Discussion

### 3.1. Morphology and Structure Characterizations of A-NiO NPs/rGO

**Figure 1:**
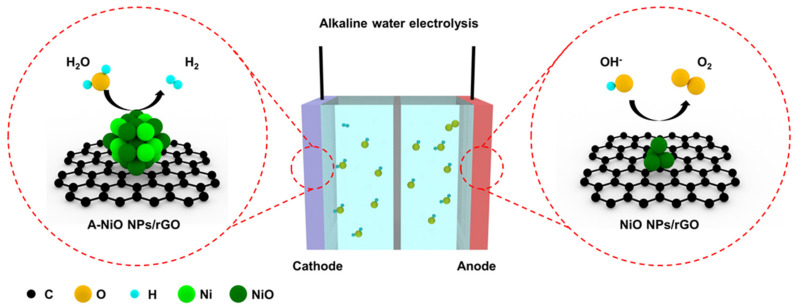
Schematic illustration of the alkaline water electrolysis, where A-NiO NPs/rGO is used for HER and NiO NPs/rGO is applied to OER. Colored spheres indicate several elements (Black: C, Yellow: O, Cyan: H, Light green: Ni, Dark green: NiO).

Figure 1 shows a schematic illustration of the overall water splitting in an alkaline electrolyte with two reactions generated at individual electrodes. At the cathode side, HER occurs, wherein a water molecule is dissociated into a hydrogen ion and a hydroxide ion, and then the hydrogen ion adsorbs on the catalyst surface. After that, the adsorbed hydrogen reacts with another water molecule or another neighboring hydrogen intermediate, generating a hydrogen molecule [32]. In alkaline media, there are only trace amounts of hydrogen ions, which could participate in HER, so that water molecules should be dissociated. For this reason, HER electrocatalysts are required to be active in the dissociation of water molecules and also have moderate energy to attach and detach hydrogen easily on their surface. In addition, at the anode side, the OER takes place, wherein a hydroxide ion adsorbs on the catalyst surface, and then another hydroxide ion participates in the reaction, thereby finally generating an oxygen molecule [33]. In the case of the OER, a hydroxide ion mainly involves in the reaction. Thus, it is necessary for the OER catalyst to interact with negatively charged hydroxide ions.

Several studies have tried to enhance the catalytic activity of both HER and OER by metal–carbon coupling, heteroatom doping, or the introduction of additional metals. However, they had significant drawbacks such as high temperature-involved or time-consuming synthesis, catalyst aggregation, and relatively large-size particles. Herein, we fabricated a few nanometer-sized NiO particles on the rGO by facile and fast microwave-assisted synthesis and controlled their interface by mild thermal annealing process under reductive atmosphere. Based on previous reports, the co-existence of metallic and oxide phases with Ni and NiO could show synergistic effects in HER for water molecule dissociation and hydrogen generation [24,25]. Moreover, single NiO would be efficient in OER for hydroxide ion adsorption [9]. From this point of view, we selectively adopted phase-controlled electrocatalysts into the cathode and the anode for overall water splitting. After preparation of the NiO NPs/rGO, the morphology and structure of the electrocatalysts were investigated by TEM.

**Figure 2:**
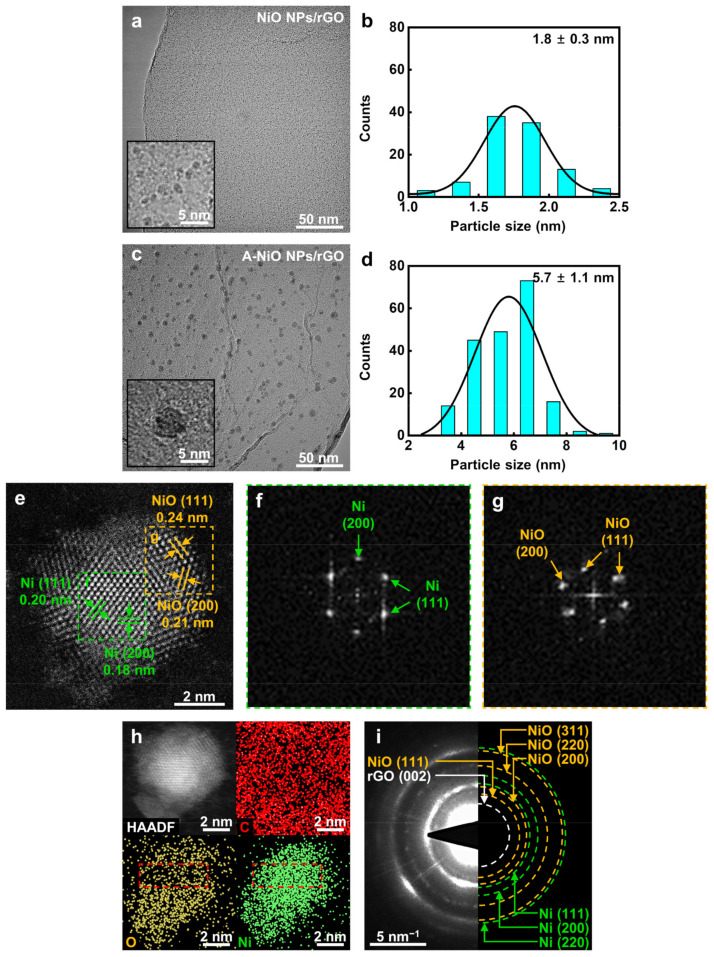
Morphology and structure characterizations. TEM images and corresponding particle size distribution of (**a**,**b**) NiO NPs/rGO and (**c**,**d**) A-NiO NPs/rGO. Insets of (**a**) and (**c**) are enlarged images. (**e**) HAADF-STEM image and (**f**,**g**) locally characterized FFT images of one Ni-NiO NP from A-NiO NPs/rGO. (**h**) HAADF-EDS mapping of the Ni-NiO NP from A-NiO NPs/rGO. (**i**) SAED image of A-NiO NPs/rGO.

As shown in Figure 2a, the particles on the synthesized NiO NPs/rGO were clearly observed in high magnification and they were uniformly dispersed on the surface of the rGO in a round shape without any agglomeration. Based on high-resolution TEM images, the size of NPs was determined to be 1.8 nm on average (see Figure 2b). Figure S1a,c and Figure 2c demonstrate the annealed NiO NPs/rGO in a reductive atmosphere at 300 °C, 400 °C, and 500 °C, respectively. As the annealing temperature elevated, the sizes of the NPs increased to 4.4, 5.7, and 9.7 nm, because smaller NPs preferred to dissolve and attach to neighboring larger particles by Ostwald ripening (see Figure S1b,d and Figure 2d) [34]. Additionally, the NPs and rGO support maintained their shapes up to 400 °C, whereas NPs were partially agglomerated and the rGO support was relatively damaged when the temperature reached 500 °C. In addition, graphene oxide (GO) was used to investigate the support material effect on electrocatalysis. The as-synthesized NiO NPs/GO and its annealed product at 400 °C, which is A-NiO NPs/GO, were also characterized by TEM with corresponding size distributions, as described in Figure S2. Similar to NiO NPs/rGO, as-synthesized NiO NPs/GO showed that monodispersed NiO NPs were anchored on the surface of the GO with an average size of 1.1 nm (see Figure S2a,b). After annealing at 400 °C, A-NiO NPs/GO formed an increased NP size of 5.1 nm (see Figure S2c,d).

To investigate the crystallinity of the NPs, we conducted XRD analyses for the NiO NPs/rGO and A-NiO NPs/rGO. However, as shown in Figure S3, there are no clear peaks either Ni or NiO for both catalysts. This is because the size of the NPs is too small to be observed, so a high angle annular dark field-scanning transmission electron microscopy (HAADF-STEM) analysis was performed for individual catalysts to verify the structure change of the NPs. The HAADF-STEM image shows multiple facets of an A-NiO NP anchored on the rGO surface (see Figure 2e). There are two distinguished areas with different crystallinity. The center of the NP (green dashed line area) shows (111) and (200) planes of Ni and the outer surface of the NP (yellow dashed line area) represents (111) and (200) planes of NiO. Moreover, fast Fourier transform (FFT) images were extracted from each area of Figure 2e. These images also support that single NP consists of separated phases of Ni (Figure 2f) and NiO (Figure 2g).

Furthermore, elemental analyses were conducted using EDS. As shown in Figure S4, the EDS spectrum of the A-NiO NPs/rGO confirms that it consists of carbon, oxygen, and nickel. To investigate spatial element distribution of the A-NiO NPs/rGO, HAADF-EDS mapping analyses were also performed (see Figure 2h). They clearly show that the NP is composed of Ni and O with almost the same distribution shape from the HAADF image of NP, and also the NP is on the carbonaceous support with the homogeneous distribution of carbon. Remarkably, we could also observe the central part of the NP (red dashed line area) with Ni-rich and O-deficient areas in the A-NiO NPs/rGO. On the other hand, Figure S5a shows that oxygen is homogeneously dispersed in the NiO NPs/rGO. We believe that this outcome is from the partial reduction of NiO NPs surface during the annealing procedure of the A-NiO NPs/rGO.

For more clarification, we collected the selected area electron diffraction (SAED) of the A-NiO NPs/rGO (Figure 2i). The SAED shows several ring-shaped patterns at the center, which could be assigned to planes of the C and Ni-related phases. The rGO (002) related pattern appears nearest to the center, and also NiO (111) related pattern is weakly detected, which is distinct from Ni (111). In addition, larger rings correspond to higher index facets of Ni and NiO, which overlapped each other but could be distinguished as green dashed ring (Ni) and yellow dashed ring (NiO), respectively. Meanwhile, clear patterns of each phase were hardly seen in the NiO NPs/rGO (see Figure S5b). To this end, we might conclude that the A-NiO NPs/rGO was synthesized in a form of two compatible phases (Ni–NiO) on the carbonaceous support (rGO).

**Figure 3:**
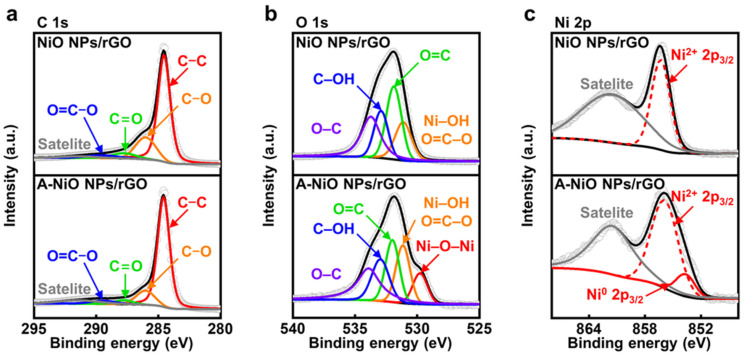
XPS characterizations for thermal annealing. XPS spectra of (**a**) C 1s, (**b**) O 1s, (**c**) Ni 2p_3/2_ for NiO NPs/rGO (upper) and A-NiO NPs/rGO (lower).

To confirm the chemical states of the NiO NPs/rGO and the A-NiO NPs/rGO, we additionally performed X-ray photoelectron spectroscopy (XPS) analyses. First of all, the C 1s fine spectra of the NiO NPs/rGO and the A-NiO NPs/rGO are shown in Figure 3a. Both samples contain five deconvoluted peaks at 284.6, 286.0, 287.8, 289.4, and 291.4 eV, which correspond to C–C, C–O, C=O, O=C–O, and π–π satellite, respectively [35]. The C–C bonding indicates carbon atoms in a conjugated lattice of the graphene layer, whereas the residual C–O, C=O, and O=C–O bondings originated from the epoxide, carbonyl, and carboxylate groups on the rGO surface. Moreover, including C–O bonding, the peak intensities of oxygen-related functional groups are decreased after annealing of the NiO NPs/rGO, because of the reduction of the rGO. In addition, the O 1s fine spectra of the NiO NPs/rGO and the A-NiO NPs/rGO commonly present four peaks at 531.2, 532.0, 533.0, and 533.9 eV, which are related to Ni–OH/O=C–O, O=C, C–OH, and O–C, respectively (see Figure 3b) [36]. Each peak reflects carboxylate, carbonyl, hydroxyl, and epoxide functional groups, which are well-matched with the outcomes of the C 1s analysis. Additionally, the Ni–OH peak is derived from adsorbed OH^−^ species on Ni^2+^ surface [37]. Here, noticeably, a new peak appeared at 529.8 eV in the case of the A-NiO NPs/rGO, which indicates lattice oxygen Ni–O–Ni [37]. It might be assumed that the crystalline Ni–O bonding was formed during the annealing process, and it is consistent with previously presented TEM data.

Finally, we compared Ni 2p fine spectra of the NiO NPs/rGO and the A-NiO NPs/rGO samples to determine the partial reduction of NiO after the annealing procedure as we observed from TEM analyses. As described in Figure 3c, both samples contain two common peaks of Ni^2+^ 2p_3/2_ and its satellite of the NiO at 856.0 and 861.5 eV, respectively [38]. On the other hand, the A-NiO NPs/rGO shows another peak at 853.6 eV, which corresponds to the Ni^0^ 2p_3/2_ state [38,39]. This outcome clearly supports the generation of metallic Ni phase after thermal reduction, and thereby, the co-existence of two phases of Ni in metallic and oxide states for the A-NiO NPs/rGO. Additionally, we also conducted XPS analyses for the NiO NPs/GO and the A-NiO NPs/GO to check the influence of the GO as a support material (see Figure S6). They show a relatively higher ratio of oxygen-containing carbon functional groups. However, except for those parts, they represent almost the same trends with the rGO used catalysts in C 1s, O 1s, and Ni 2p peaks before and after the annealing process.

### 3.2. Electrochemical Measurements of the Catalysts

**Figure 4:**
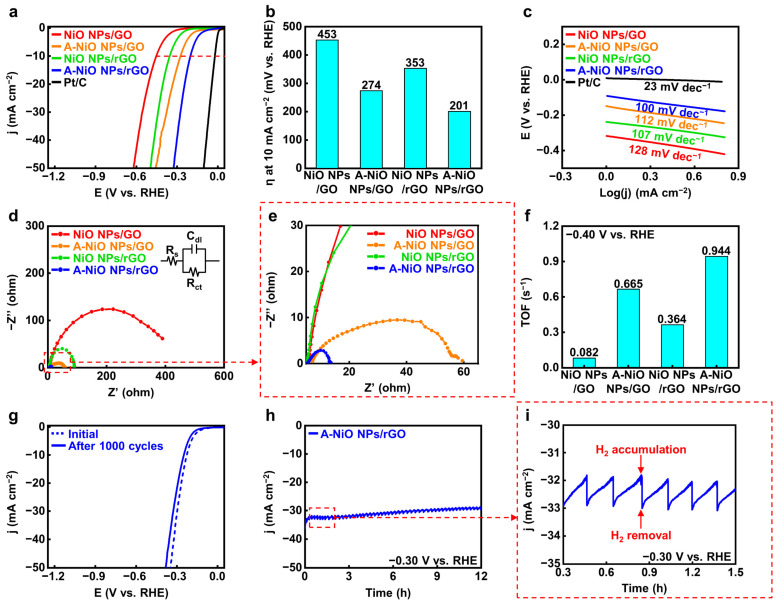
Electrochemical measurement for HER. (**a**) LSV curves for HER and (**b**) corresponding overpotentials at 10 mA cm^−2^ of NiO NPs/GO, A-NiO NPs/GO, NiO NPs/rGO, A-NiO NPs/rGO, and Pt/C in 1 M KOH at a scan rate of 5 mV s^−1^. (**c**) Tafel slopes of NiO NPs/GO, A-NiO NPs/GO, NiO NPs/rGO, A-NiO NPs/rGO, and Pt/C. (**d**) EIS curves and (**e**) enlarged data of NiO NPs/GO, A-NiO NPs/GO, NiO NPs/rGO, and A-NiO NPs/rGO at an overpotential of −0.30 V vs RHE. Inset of (**d**) is the Randles circuit of the HER. (**f**) Histograms for TOF of NiO NPs/GO, A-NiO NPs/GO, NiO NPs/rGO, and A-NiO NPs/rGO at −0.40 V vs. RHE. (**g**) LSV curves of A-NiO NPs/rGO before and after 1000 cycles of durability test. (**h**) CA test of A-NiO NPs/rGO under an overpotential of −0.30 V vs. RHE for 12 h. (**i**) Zoomed in data from (**h**).

After proving the co-existence of oxide and metallic phases in Ni, we carried out electrochemical analyses for the A-NiO NPs/rGO with a three-electrode system in 1 M KOH aqueous solution to evaluate the catalytic activity and durability of the HER performance. Figure 4a shows the LSV curves of the catalysts, and Figure 4b represents their overpotentials at 10 mA cm^−2^ of current density (red dashed line) from the curves. In the case of the NiO NPs/rGO, 353 mV is required to achieve 10 mA cm^−2^, while the A-NiO NPs/rGO shows much lower overpotential as 201 mV, the lowest value compared to that of previously studied catalysts, which are from 205 mV to 410 mV vs. RHE (see Table S2). This might originate from the synergistic effect of the Ni-NiO dual-phase. Furthermore, the NiO NPs/GO and the A-NiO NPs/GO samples exhibit 453 mV and 274 mV, respectively. Here, the annealed sample has a lower overpotential value than the as-synthesized sample, because of partial reduction of the NiO NPs. In general, the GO-supported materials have relatively higher overpotential values than that of rGO-supported materials and annealed samples with the same supporting materials have lower overpotential values than that of as-synthesized samples.

For detailed elucidation of the correlation between the catalytic activities and the annealing conditions, we carried out LSV for different annealing temperatures and different atmospheres. We determined that all of the annealed catalysts outperform the as-synthesized NiO NPs/rGO (see Figure S7a,b). The annealing temperature has an optimal HER activity at 400 °C, implying the deterioration of performance from the aggregation of NPs and the decrease in active surface area on NPs by Ostwald ripening at the elevated temperature such as 500 °C (see Figure S1c,d). Furthermore, as shown in Figure S8a,b, we found that thermal annealing without H_2_ showed slightly higher overpotential (243 mV) than A-NiO NPs/rGO, suggesting that hydrogen precursor partially reduces NiO NPs (see Figure 3c), and it affects the catalytic activity in HER. Additionally, Tafel slopes of the same materials confirm that the A-NiO NPs/rGO has the lowest value among Ni-based catalysts, 100 mV dec^−1^. This implies that hydrogen adsorption and desorption on the surface of the A-NiO NPs/rGO affects the reaction rate more than water molecule dissociation. Meanwhile, the NiO NPs/GO (128 mV dec^−1^), A-NiO NPs/GO (112 mV dec^−1^), and NiO NPs/rGO (107 mV dec^−1^) showed higher slopes than the A-NiO NPs/rGO, suggesting that water dissociation still affects the whole reaction rate in HER, showing sluggish hydrogen evolution compared to the A-NiO NPs/rGO at the same potential (see Figure 4c). Furthermore, EIS analyses were performed to compare the charge transfer rates of the catalysts. There are three components that constitute a Randle circuit, as shown in the inset of Figure 4d [40]. The solution resistance (R_s_) was measured at a high frequency of 100 kHz, and double-layer capacitance (C_dl_) appeared in the form of a semicircle as the frequency decreased. The charge transfer resistance (R_ct_) was estimated at a low frequency of 0.01 Hz. More specifically, the catalyst has a fast charge transfer at the interface when it has a low R_ct_ under constant R_s_. The R_ct_ values of the catalysts are displayed in Figure 4d,e and the NiO NPs/GO, A-NiO NPs/GO, NiO NPs/rGO, and A-NiO NPs/rGO are approximately 400, 55, 85, and 10 Ω, respectively. Thus, the A-NiO NPs/rGO exhibits the lowest charge transfer resistance among these materials, which suggests that a faster chemical reaction could be achieved at the catalyst interface. In addition, TOF was calculated at −0.40 V vs. RHE in order to evaluate HER efficiency, as described in Figure 4f. For the TOF calculation, the amount of Ni was determined by using ICP-OES, as shown in Table S1. We found that the weight ratios of each catalyst were 6.7, 8.7, 7.9, and 14.2 wt % for the NiO NPs/GO, A-NiO NPs/GO, NiO NPs/rGO, and A-NiO NPs/rGO, respectively. The A-NiO NPs/rGO has the fastest rate of switching from hydrogen atoms to hydrogen molecules on its surface, with 0.944 s^−1^. In addition, the results indicate that active materials on the rGO have higher activity than when they are on the GO because oxygen-containing functional groups on the graphene support interfere with the conduction of electrons during the chemical reaction. It is well-matched with the results of the HER performances, which are described in Figure 4a. Furthermore, we estimated the mass-normalized catalytic activity by ICP-OES and LSV. Although the quantitative amount of the metal NPs was different for each catalyst, HER activity shows a consistent result, as displayed in Figure S9a, indicating that A-NiO NPs/rGO exhibits the best performance among the non-precious metal-anchored catalysts.

For further characterization, we evaluated the long-term durability of CV and CA. In Figure 4g, the LSV curve of the A-NiO NPs/rGO shows a negligible change between the initial state (blue dashed line) and after 1000 cycles curves (blue solid line), indicating that its catalytic activity was maintained in spite of repeated and prolonged redox reactions. Moreover, the TEM image and particle size distribution in Figure S10a,b show that the NPs are rarely aggregated or detached from rGO surface. This indicates that the A-NiO NPs/rGO maintained its morphology after the durability test. We also investigated XPS for A-NiO NPs/rGO after the CV test, and found that both nickel and nickel oxide peaks are maintained after the durability test, as displayed in Figure S11. This means that two distinct phases are negligibly changed during the repeated redox reaction, confirming that A-NiO NPs/rGO has superior durability. Furthermore, CA data confirm its notable stability in alkaline media at −0.30 V vs. RHE for over 12 h (see Figure 4h). Moreover, during CA measurement, we observed that hydrogen bubbles occurred on the GCE surface, which would block the interaction between the GCE surface and the electrolyte. The bubbles accumulated on the surface of GCE and burst, after then, the catalyst could contact the liquid electrolyte again for hydrogen generation. To this end, the graph has repeated zigzag patterns, as displayed in Figure 4i.

**Figure 5:**
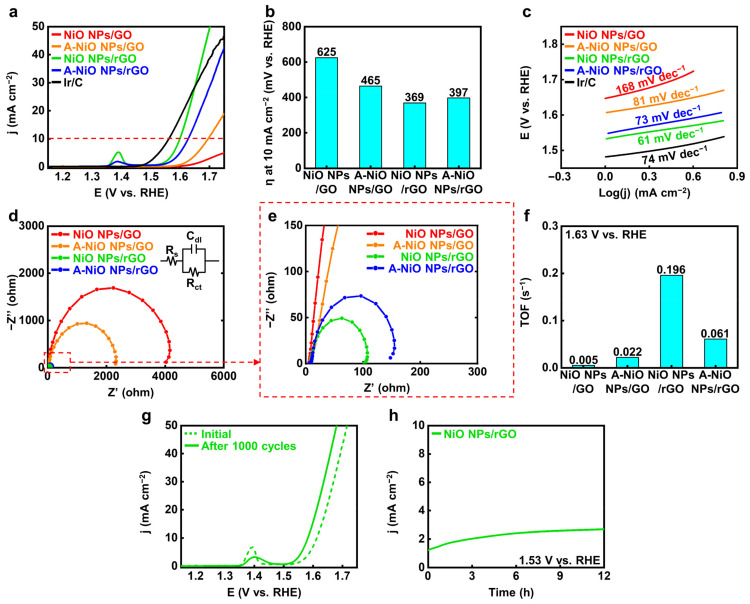
Electrochemical measurements for OER. (**a**) LSV curves for OER and (**b**) corresponding overpotentials at 10 mA cm^−2^ of NiO NPs/GO, A-NiO NPs/GO, NiO NPs/rGO, A-NiO NPs/rGO, and Ir/C in 1 M KOH at a scan rate of 5 mV s^−1^. (**c**) Tafel slopes of NiO NPs/GO, A-NiO NPs/GO, NiO NPs/rGO, A-NiO NPs/rGO, and Ir/C. (**d**) EIS curves and (**e**) enlarged data of NiO NPs/GO, A-NiO NPs/GO, NiO NPs/rGO, and A-NiO NPs/rGO at an overpotential of 1.53 V vs RHE. Inset of (**d**) is the Randles circuit of the OER. (**f**) Histograms for TOF of NiO NPs/GO, A-NiO NPs/GO, NiO NPs/rGO, and A-NiO NPs/rGO at 1.63 V vs. RHE. (**g**) LSV curves of A-NiO NPs/rGO before and after 1000 cycles for durability test. (**h**) CA test of A-NiO NPs/rGO under an overpotential of 1.53 V vs. RHE for 12 h.

We also evaluated the OER performances of the catalysts compared to commercial Ir/C. Figure 5a shows the LSV curves of the catalysts and Figure 5b represents their corresponding overpotentials at 10 mA cm^−2^ (red dashed line), respectively. As a reference sample, the commercial Ir/C represents 332 mV as the overpotential. The NiO NPs/rGO exhibits the lowest overpotential as 369 mV compared to those of the A-NiO NPs/rGO as 397 mV, the A-NiO NPs/GO as 465 mV, and the NiO NPs/GO as 625 mV. It shows higher OER activity than previously reported catalysts, ranging from 390 mV to 510 mV vs. RHE (see Table S3). Additionally, small peaks near 1.40 V vs. RHE occur for the NiO NPs/rGO and the A-NiO NPs/rGO. These peaks are originated from the change in NiO to Ni(OH)_2_ when exposed to the alkaline solution, and Ni(OH)_2_ is oxidized to NiOOH in the anodic potential [41]. Moreover, it implies that if the area of the peak becomes larger, the surface area of the catalyst will be increased, and thus, it will have a higher number of active sites in OER [42]. In fact, the NiO NPs/rGO shows the largest peak among all the measured catalysts, and it also matches well with the aforementioned results. Furthermore, as shown in Figure 5c, NiO NPs/rGO exhibits the lowest Tafel slope with 61 mV dec^−1^, which is lower than those of the A-NiO NPs/rGO with 73 mV dec^−1^, the A-NiO NPs/GO with 81 mV dec^−1^, the NiO NPs/GO with 168 mV dec^−1^, and the Ir/C with 74 mV dec^−1^. It also represents that hydroxyl ions are prone to adsorb on the NiO NPs/rGO surface and trigger oxygen evolution faster than other catalysts, resulting in less overpotential to achieve specific current density. Based on this outcome, we might regard that the NiO NPs/rGO has superior OER performance to all of the synthesized electrocatalysts. In addition, we conducted EIS measurements to compare the charge transfer resistance on the catalyst surface, as shown in Figure 5d,e. We found that the NiO NPs/rGO has the lowest R_ct_ with approximately 100 Ω, and the resistance increases in the cases of the A-NiO NPs/rGO with 150 Ω, the A-NiO NPs/GO with 2300 Ω, and the NiO NPs/GO with 4000 Ω. These results indicate that the NiO NPs/rGO has surpassing charge transfer characteristics on the surface. Accordingly, the NiO NPs/rGO demonstrates the highest oxygen turnover rate, as shown in Figure 5f. The TOF calculation for OER also confirms that the NiO NPs/rGO could convert hydroxyl ions to oxygen molecules rapidly under a given potential of 1.63 V vs. RHE. In addition, we also measured the LSV of the catalysts, which are annealed at different temperatures, and it could be considered that the annealing procedure would not promote OER activity (see Figure S7c,d), and thermal annealing without H_2_ shows better activity than H_2_-introduced A-NiO NPs/rGO (see Figure S8c,d). Moreover, mass-normalized activity in OER also corresponds with the geometrically normalized data, as shown in Figure S9b. For further characterization, we analyzed the long-term durability of the OER by CV and CA. As shown in Figure 5g, an LSV curve after 1000 cycles (green solid line) exhibits a decreased peak at 1.40 V vs. RHE, but a slightly higher activity than the initial curve (green dashed line), indicating that its stability was maintained through repeated redox reactions. The CA graph in Figure 5h shows that the NiO NPs/rGO retained its activity over 12 h at 1.53 V vs. RHE, which suggests its high durability performance in an alkaline solution.

### 3.3. Correlation between the Reaction Mechanism and Electrochemical Performances

The electrochemical results on the HER and OER could be more specifically explained by the mechanisms described in Figure S12. In the case of the HER in alkaline media, it is necessary to dissociate water molecules because of hydrogen intermediates that participate in the generation of hydrogen molecules. The water molecules are separated into hydroxide ions and hydrogen ions due to the presence of NiO, and then hydrogen species are adsorbed on the metallic Ni surface. This step is called the Volmer step [25,43]. As the next step, hydrogen molecules could be generated from the adsorbed hydrogen species in two ways. One way is called the Heyrovsky step, where the adsorbed hydrogens, water molecules, and electrons could react together to produce gaseous H_2_ and hydroxide ions [44]. The other way is called the Tafel step, where the interaction of two adsorbed hydrogen intermediates could form H_2_ molecules [45]. The phases of Ni that take the lead in each reaction are separated, which implies that the reaction rate could be improved when two metallic and oxide Ni phases could co-exist. More precisely, it is harder for H^+^ ions to attach and detach on the catalyst if there is only NiO, whereas water molecules could not dissociate well when only metallic Ni is present. To this end, the partially reduced NiO is preferred for the high activity of HER. On the other hand, negatively charged species such as hydroxyl ions and oxygen-based intermediates are mainly involved in OER, as shown in Figure S13 [24]. Thus, it is more favorable to use NiO than metallic Ni due to electrostatic attraction between the species and Ni^2+^. Moreover, small-size NPs have a higher active surface area, resulting in better performance in OER. Based on these mechanisms, NiO NPs could be utilized in both reactions by controlling their interface.

### 3.4. Overall Water Splitting

**Figure 6:**
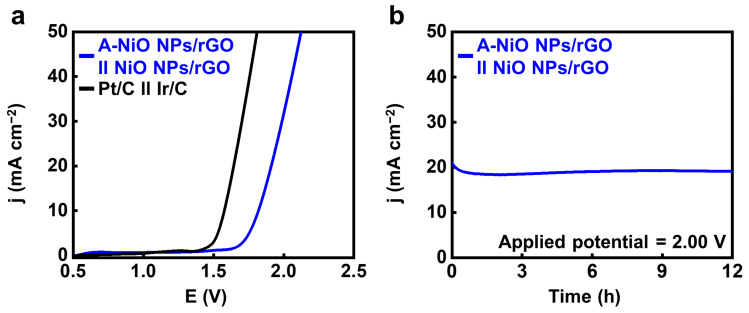
Overall water splitting test. (**a**) LSV curves of A-NiO NPs/rGO ‖ NiO NPs/rGO and Pt/C ‖ Ir/C in 1 M KOH at 5 mV s^−1^ in a two-electrode system. (**b**) CA data for long-term durability at an applied potential of 2.00 V for 12 h.

Finally, we performed a full-cell test using a two-electrode cell configuration in 1 M KOH with the A-NiO NPs/rGO as the negative electrode and the NiO NPs/rGO as the positive electrode by coating the catalyst ink on the Ni foam. For comparison, Pt/C and Ir/C were used as the cathode and anode under the same conditions, respectively. As shown in Video S1, oxygen bubbles are generated from the anode coated with NiO NPs/rGO (left side), and hydrogen bubbles are released from the cathode coated with A-NiO NPs/rGO (right side). Figure 6a shows that noble metal-based catalysts have ~1.58 V of potential at 10 mA cm^−2^, whereas the A-NiO NPs/rGO in the cathode and the NiO NPs/rGO in the anode demonstrates ~1.82 V at the same condition. In addition, the long-term durability of our non-precious catalysts was evaluated for 12 h under a constant potential of 2.00 V (see Figure 6b). It was observed that the current density remained almost unchanged while maintaining approximately 20 mA cm^−2^ during measurement, confirming its stability for overall water splitting.

## 4. Conclusions

In summary, we synthesized NiO NPs on the rGO surface by the facile microwave-assisted method and controlled their interface through thermal reduction. The surface of NPs changed from oxidized NiO to partially metallic Ni, resulting in two distinct co-existing Ni-NiO phases. It promoted reaction rates for hydrogen evolution in alkaline media by showing synergistic effects, including that NiO facilitates dissociation of water molecules and Ni takes part in hydrogen adsorption/desorption. On the other hand, only NiO was beneficial to OER due to the interaction between the catalyst and negatively charged reactants. The TEM and XPS results demonstrated the change of the surface state of the NPs with the appearance of the metallic Ni phase. Moreover, electrochemical measurements could support the previously mentioned theoretical mechanism. The A-NiO NPs/rGO had 201 mV to reach a current density of 10 mA cm^−2^ in HER, which is much lower than NiO NPs/rGO (353 mV). On the other hand, as-synthesized NiO NPs/rGO could achieve only 369 mV of overpotential under the same current density, which is higher activity than that of the A-NiO NPs/rGO (397 mV) in OER. Furthermore, the A-NiO NPs/rGO maintained its morphology and structure over 1000 repeated redox cycles and under constant potential for 12 h. Finally, we applied each catalyst selectively into an overall water splitting system and confirmed their full cell capability. From these results, the A-NiO NPs/rGO and NiO NPs/rGO could be possible candidates to replace precious metal-based electrocatalysts. This research could promote practical applications of alkaline water electrolysis technology.

## Data Availability

Data are contained within the article or Supplementary Materials.

## Supplementary Materials

The following are available online at https://www.mdpi.com/article/10.3390/nano11123379/s1, TOF calculation, Figure S1: TEM images and corresponding particle size distribution of (a,b) A-NiO NPs/rGO (300°C) and (c,d) A-NiO NPs/rGO (500°C). Inset images of (a) and (c) are enlarged views, respectively. Figure S2: TEM images and corresponding particle size distribution of (a,b) NiO NPs/GO and (c,d) A-NiO NPs/GO. Inset images of (a) and (c) are enlarged views, respectively. Figure S3: XRD spectra of the NiO NPs/rGO and A-NiO NPs/rGO. Figure S4: (a) EDS spectrum of A-NiO NPs/rGO, (b) enlarged view of (a). Detected Cu peaks were originated from the Cu grid. Figure S5: (a) EDS mapping and (b) SAED image of NiO NPs/rGO. Figure S6: XPS spectra of (a) C 1s, (b) O 1s, (c) Ni 2p_3/2_ for NiO NPs/GO (upper) and A-NiO NPs/GO (lower). Figure S7: LSV curves and corresponding overpotential histograms at 10 mA cm^−2^ in (a,b) HER and (c,d) OER with different annealing temperature. Figure S8: LSV curves and corresponding overpotential histograms at 10 mA cm^−2^ in (a,b) HER and (c,d) OER with different annealing atmosphere. Figure S9: LSV curves of the catalysts which are mass-normalized in (a) HER and (b) OER. Figure S10. (a) TEM image and (b) corresponding particle size distribution of A-NiO NPs/rGO after durability test. Figure S11. XPS spectra of Ni 2p_3/2_ for A-NiO NPs/rGO at an initial state (upper) and after durability test (lower). Figure S12: Schematic illustration of HER mechanism for Ni species in alkaline media. Figure S13: Schematic illustration of OER mechanism for Ni species in alkaline media. Table S1: ICP-OES data of catalysts. Table S2: Collected data of transition metal-carbonaceous support hybrid catalysts for HER. Table S3: Collected data of transition metal-carbonaceous support hybrid catalysts for OER. Video S1: Overall water splitting and gas generation by NiO NPs/rGO at the anode and A-NiO NPs/rGO at the cathode.
