# Supplementary material for: Phase-Controlled NiO Nanoparticles on Reduced Graphene Oxide as Electrocatalysts for Overall Water Splitting
Source: Nanomaterials (Basel). 2021 Dec 13;11(12):3379. doi: 10.3390/nano11123379 (PMC8708175; doi:10.3390/nano11123379)
Supplement: Supplementary file 1 [file nanomaterials-11-03379-s001.zip › nanomaterials-1491200 - Supplementary Materials.pdf]

nanomaterials

MDPI

Supplementary Materials

Phase-Controlled NiO Nanoparticles on Reduced Graphene Oxide as Electrocatalysts for Overall Water Splitting

Seung Geun Jo [1], Chung-Soo Kim [2,*], Sang Jun Kim [1,3,*] and Jung Woo Lee [1,*]

[1] Department of Materials Science and Engineering, Pusan National University, Busan 46241, Korea; linkroot1128@pusan.ac.kr
[2] Analysis & Certification Center, Korea Institute of Ceramic Engineering & Technology, Jinju 52851, Korea
[3] Institute of Materials Technology, Pusan National University, Busan 46241, Korea
* Correspondence: cskim@kicet.re.kr (C.-S.K.); ksj0125@pusan.ac.kr (S.J.K.); jungwoolee@pusan.ac.kr (J.W.L.); Tel.: +82-51-510-2898 (J.W.L.)

TOF calculation

Three theoretical assumptions were applied for the turnover frequency (TOF) calculation as follows: (1) The diameter of the Ni atoms is 0.24 nm. (2) Ni atoms are stacked in the form of a close-packed face-centered cubic structure. (3) Only the surfaces of the particles participated in the HER and OER. The calculation formula for the TOF is as follows:

$$TOF = \frac{number\ of\ total\ hydrogen\ turnover\ per\ cm^2}{number\ of\ active\ sites\ per\ cm^2} = \frac{N_{H_2} \times |current\ density|}{active\ sites}$$

In HER, TOF is defined as the total hydrogen turnover number per the number of active sites. Also, two electrons are involved in the reaction. Therefore, the number of total hydrogen turnovers was calculated using the following formula:

$$N_{H_2} = \left(j\frac{mA}{cm^2}\right)\left(\frac{\frac{1C}{s}}{1000\ mA}\right)\left(\frac{1\ mol\ e^-}{96485.3\ C}\right)\left(\frac{1\ mol\ H_2}{2\ mol\ e^-}\right)\left(\frac{6.023\ \times 10^{23}\ molecules\ H_2}{1\ mol\ H_2}\right)$$
$$= 3.12 \times 10^{15}\frac{H_2/s}{cm^2} per \frac{mA}{cm^2}$$

In OER, four electrons participate in the reaction, so the equation is as follows:

$$N_{O_2} = \left(j\frac{mA}{cm^2}\right)\left(\frac{\frac{1C}{s}}{1000\ mA}\right)\left(\frac{1\ mol\ e^-}{96485.3\ C}\right)\left(\frac{1\ mol\ O_2}{4\ mol\ e^-}\right)\left(\frac{6.023\ \times 10^{23}\ molecules\ O_2}{1\ mol\ O_2}\right)$$
$$= 1.56 \times 10^{15}\frac{O_2/s}{cm^2} per \frac{mA}{cm^2}$$

The number of Ni atoms was calculated from the ICP-OES data using the following formula:

$$N_{Ni} = \left(\frac{2.83 \times 10^{-4}\,\frac{g}{cm^2} \times m\,\%\,(Ni)}{58.69\,\frac{g}{mol}}\right)\left(6.023 \times 10^{23}\,\frac{Ni\ atom}{mol}\right) = 2.9 \times 10^{18} \times m\,\%\,Ni\ atoms\ per\ cm^2$$

Finally, the number of active sites was calculated as follows:

$$Active\ sites = 2.9 \times 10^{18} \times m\,\%\,Ni\ atoms\ per\ cm^2 \times \frac{The\ number\ of\ surface\ Ni\ atoms}{The\ number\ of\ Ni\ atoms}$$

From these equations, TOF can be obtained.

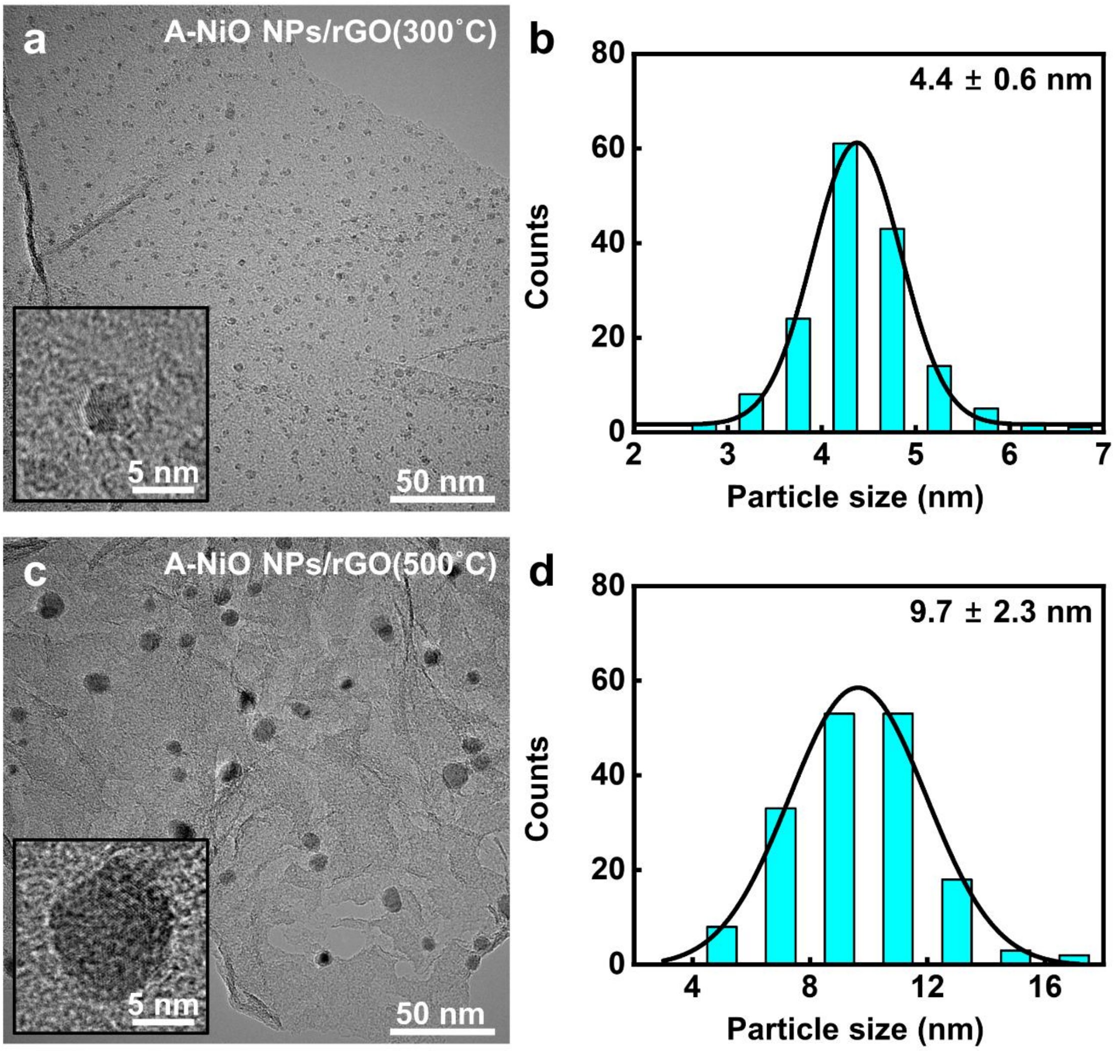

Figure S1. TEM images and corresponding particle size distribution of (**a**,**b**) A-NiO NPs/rGO (300°C) and (**c**,**d**) A-NiO NPs/rGO (500°C). Insets of (**a**) and (**c**) are enlarged images, respectively.

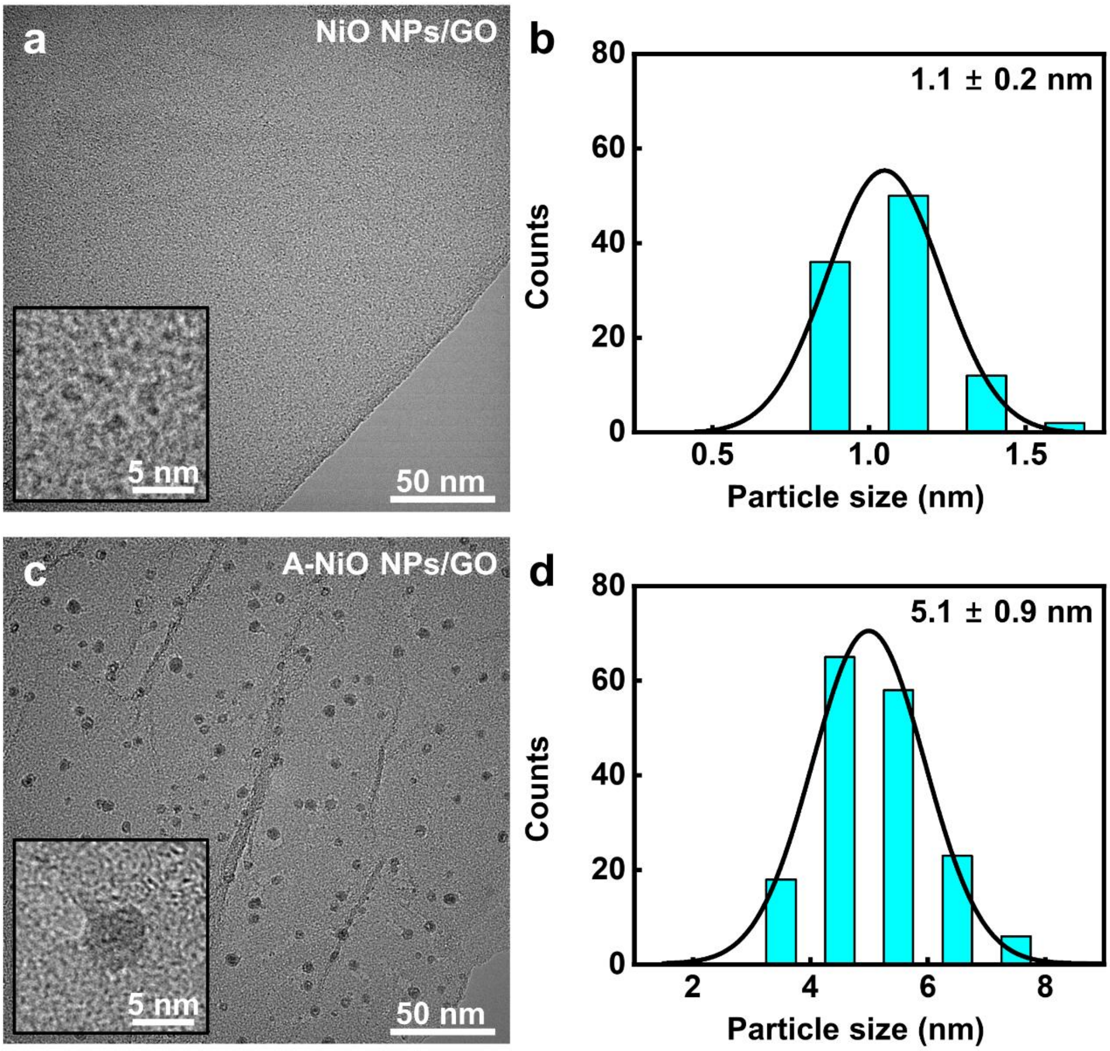

Figure S2. TEM images and corresponding particle size distribution of (**a**,**b**) NiO NPs/GO and (**c**,**d**) A-NiO NPs/GO. Insets of (**a**) and (**c**) are enlarged images, respectively.

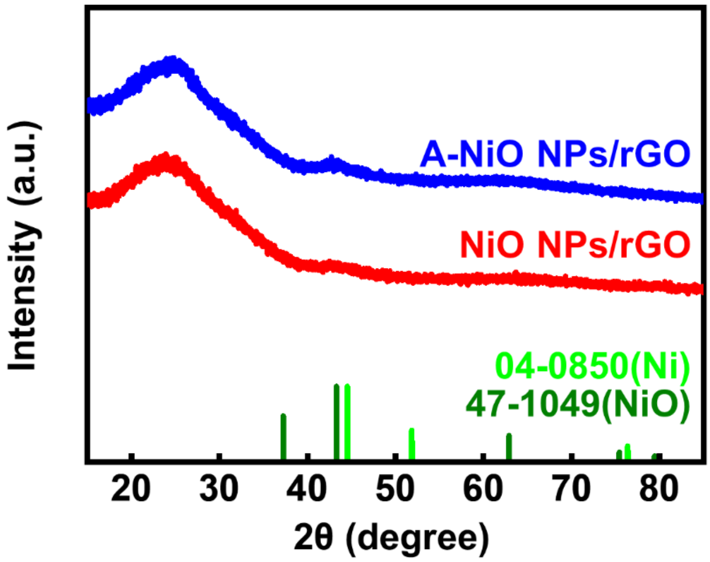

Figure S3. XRD spectra of the NiO NPs/rGO and A-NiO NPs/rGO.

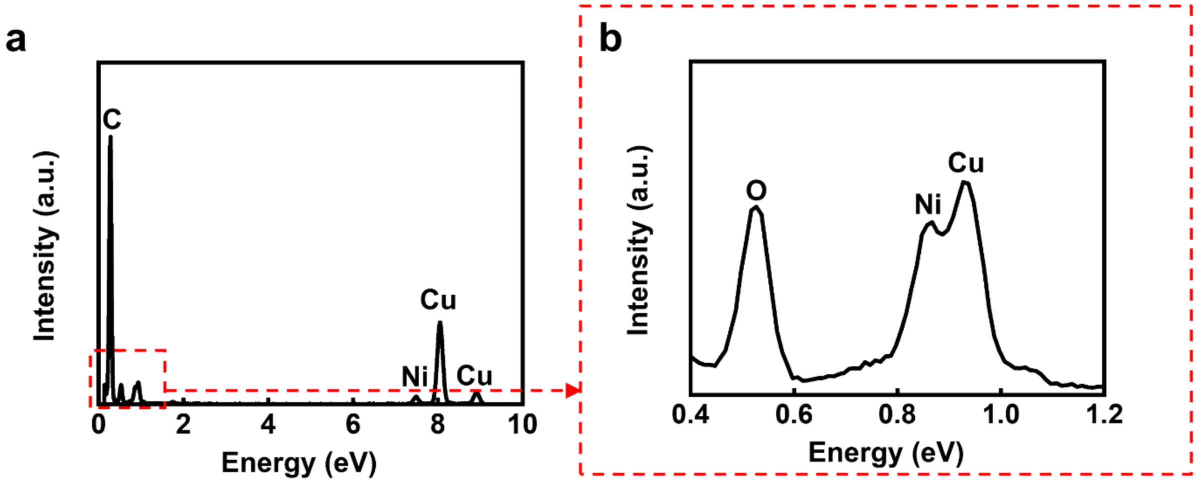

Figure S4. (**a**) EDS spectrum of A-NiO NPs/rGO, (**b**) enlarged view of (**a**). Detected Cu peaks were originated from the Cu grid.

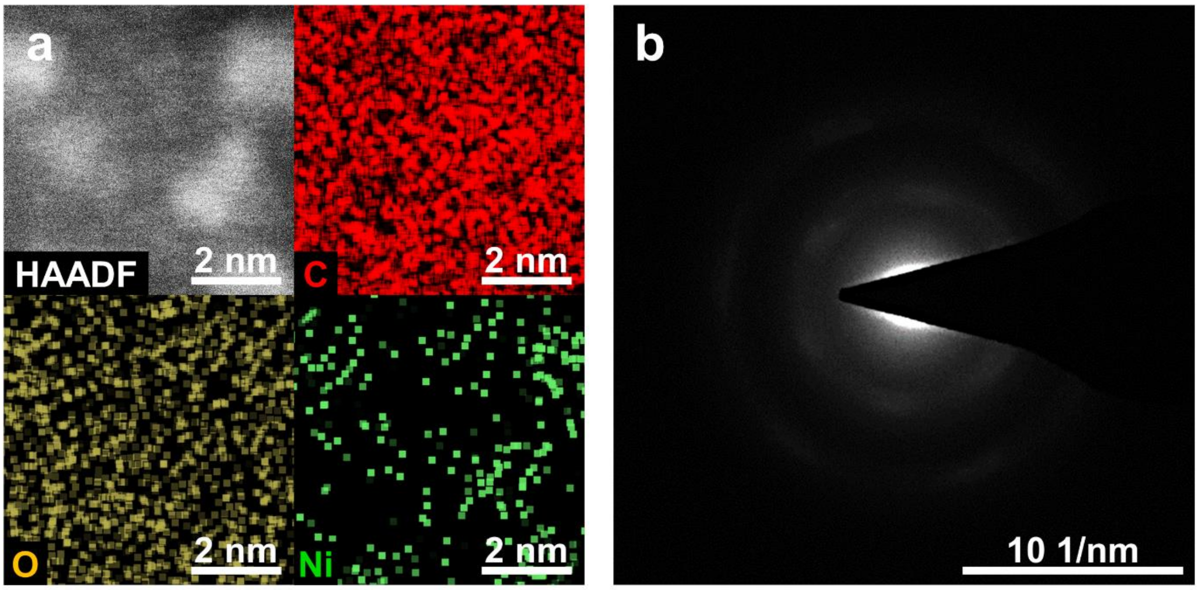

Figure S5. (**a**) EDS mapping and (**b**) SAED image of NiO NPs/rGO.

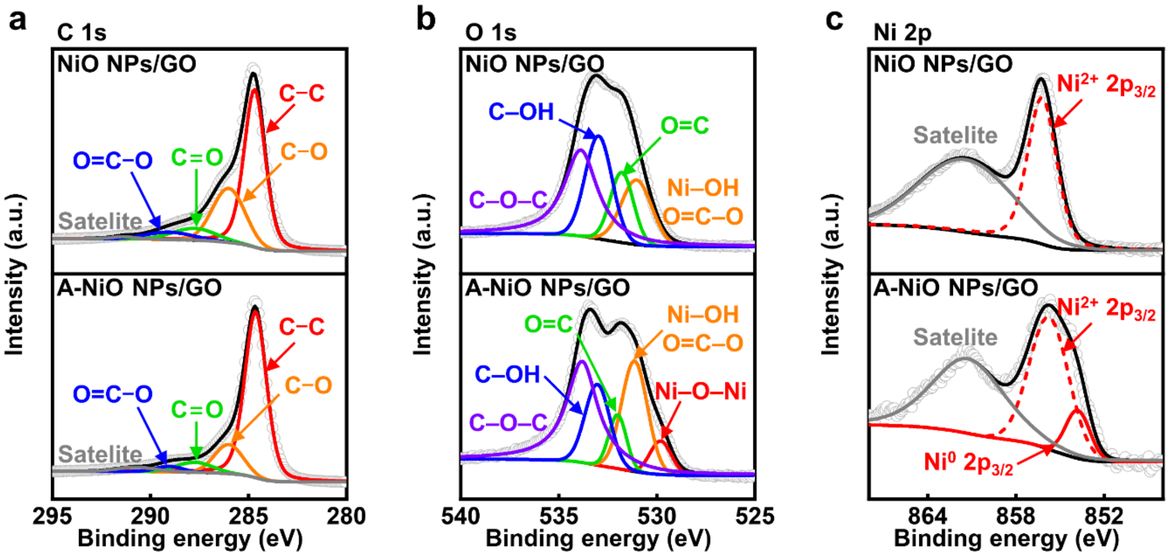

Figure S6. XPS spectra of (**a**) C 1s, (**b**) O 1s, (**c**) Ni $2p_{3/2}$ for NiO NPs/GO (upper) and A-NiO NPs/GO (lower).

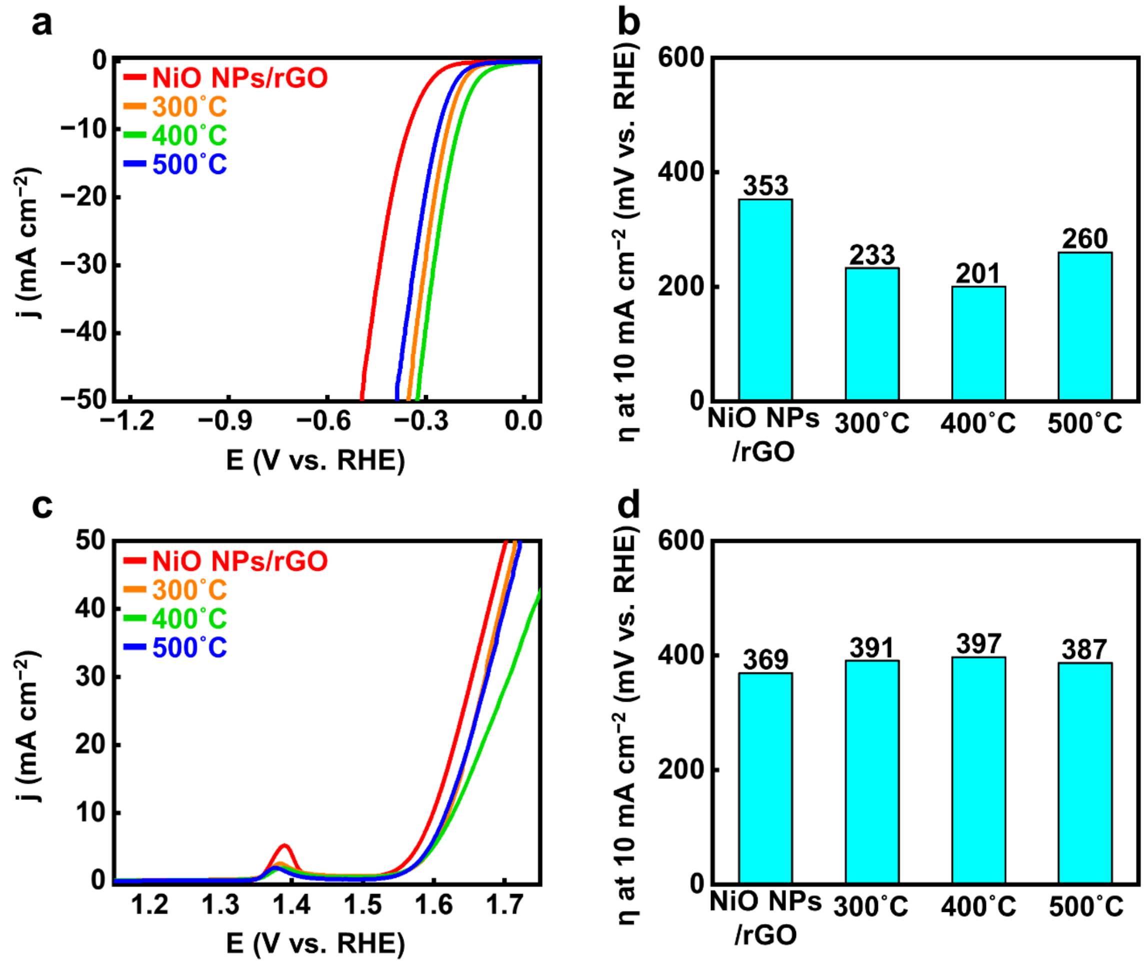

Figure S7. LSV curves and corresponding overpotential histograms at 10 mA cm^{-2} in (**a**,**b**) HER and (**c**,**d**) OER with different annealing temperature.

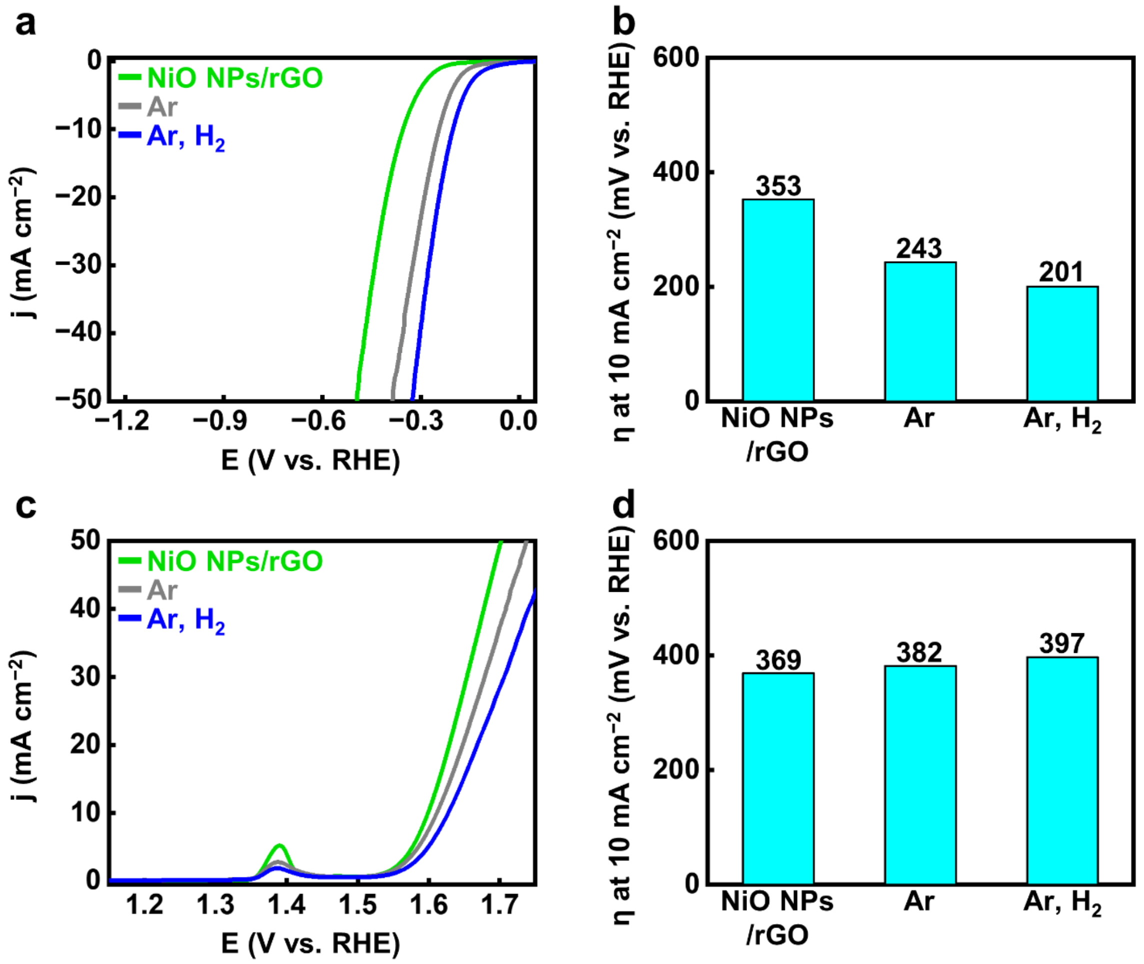

Figure S8. LSV curves and corresponding overpotential histograms at 10 mA cm^{-2} in (**a**,**b**) HER and (**c**,**d**) OER with different annealing atmosphere.

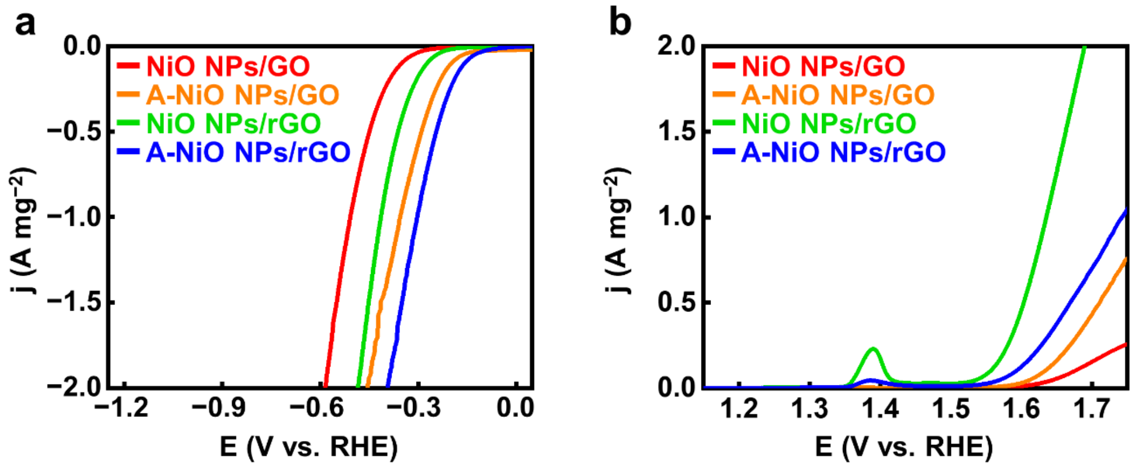

Figure S9. LSV curves of the catalysts which are mass-normalized in (**a**) HER and (**b**) OER.

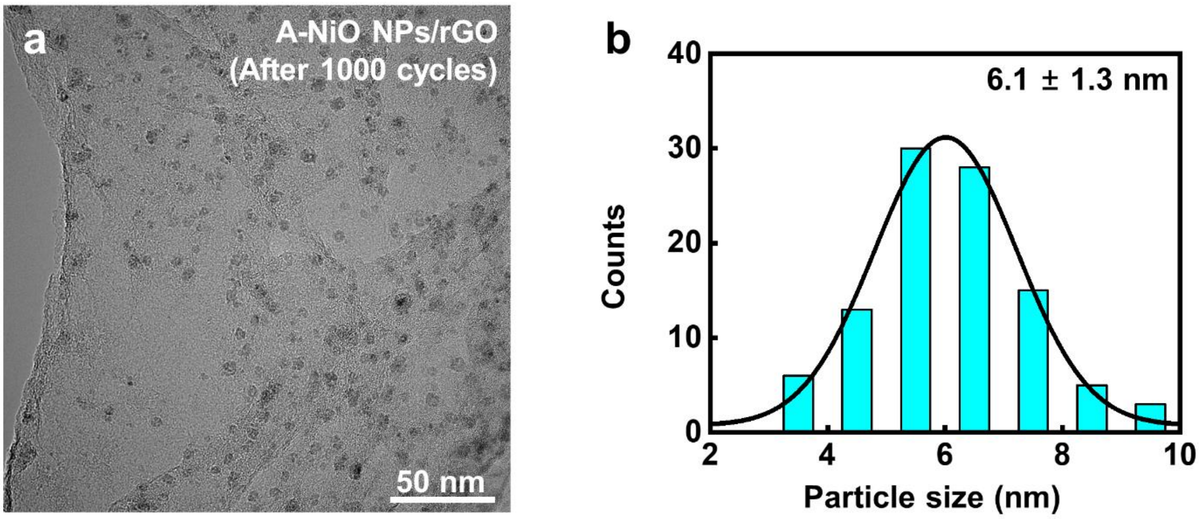

Figure S10. (**a**) TEM image and (**b**) corresponding particle size distribution of A-NiO NPs/rGO after durability test.

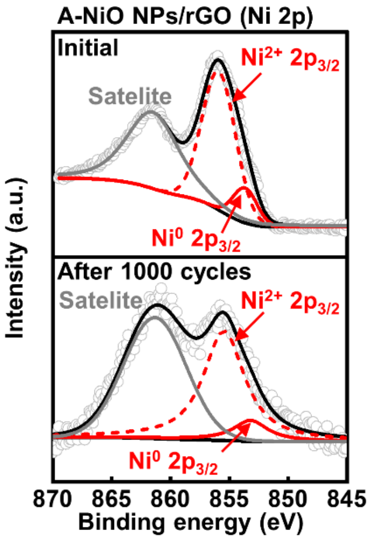

Figure S11. XPS spectra of Ni $2p_{3/2}$ for A-NiO NPs/rGO at an initial state (upper) and after durability test (lower).

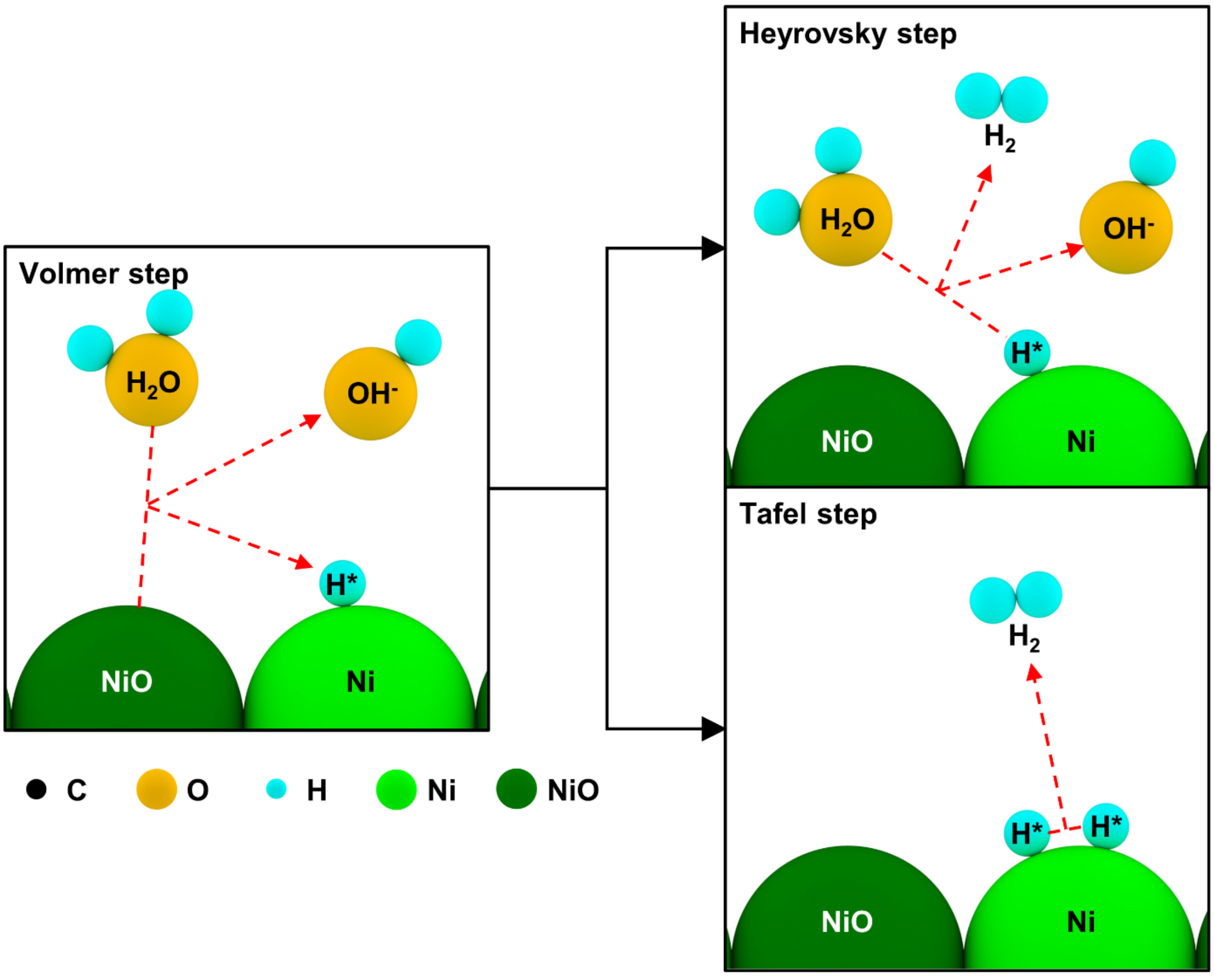

Figure S12. Schematic illustration of HER mechanism for Ni species in alkaline media.

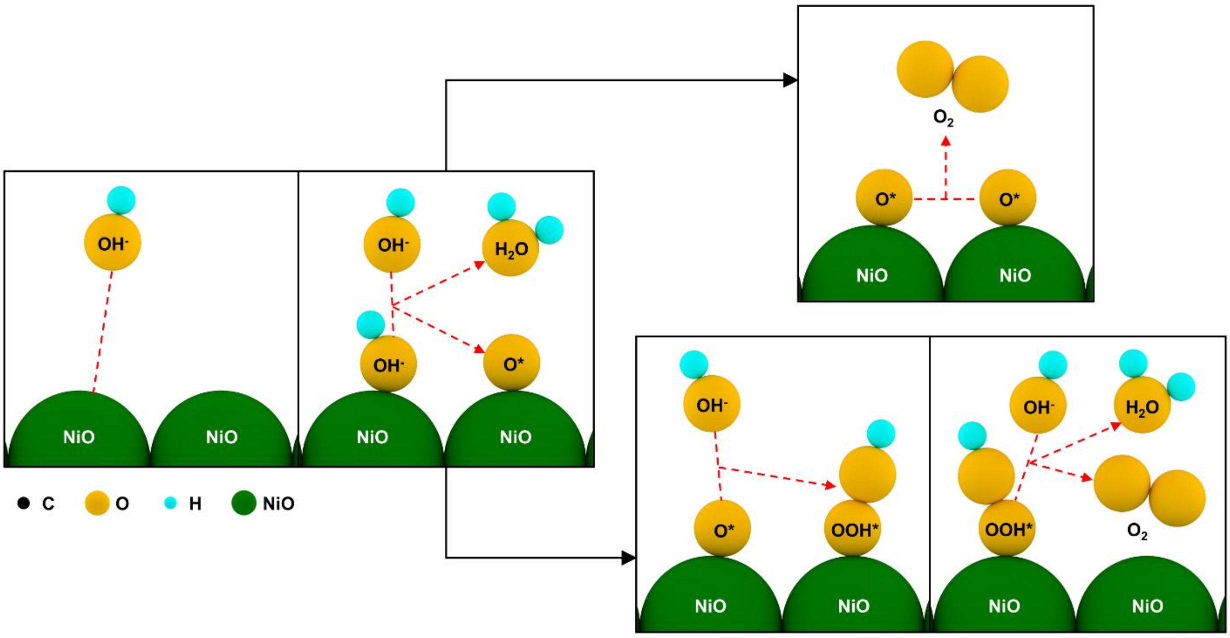

Figure S13. Schematic illustration of OER mechanism for Ni species in alkaline media.

Table S1. ICP-OES data of catalysts.

Catalysts	Loading Amount of Ni (wt%)
NiO NPs/GO	6.7
A-NiO NPs/GO	8.7
NiO NPs/rGO	7.9
A-NiO NPs/rGO	14.2

Table S2. Collected data of transition metal-carbonaceous support hybrid catalysts for HER.

Catalysts	Mass Loading (mg cm^{-2})	Electrolyte	Current Density (mA cm^{-2})	Overpotential (mV)	Ref.
A-NiO NPs/rGO	0.28	1 M KOH	10	201	This work
Co-NG	0.28	1 M NaOH	10	270	[1]
Ni@C-400	2.35	1 M KOH	10	270	[2]
Ni@NC	0.35	1 M KOH	10	205	[3]
Ni@graphene	0.35	1 M KOH	10	240	[4]
Ni/NiO@HGP$_x$O$_y$	1.1	1 M KOH	10	205	[5]
Ni/NGr	-	0.1 M KOH	10	410	[6]
Ni@CNT	0.36	1 M KOH	10	266	[7]
N-Co@G	0.28	0.5 M H_2SO_4	10	265	[8]
Ni/C/MoS_2	0.26	0.5 M H_2SO_4	10	275	[9]
Ni-NCNTs	0.25	0.5 M H_2SO_4	10	378	[10]

Table S3. Collected data of transition metal-carbonaceous support hybrid catalysts for OER.

Catalysts	Mass Loading (mg cm^{-2})	Electrolyte	Current Density (mA cm^{-2})	Overpotential (mV)	Ref.
NiO NPs/rGO	0.28	1 M KOH	10	369	This work
Doped MnO_2 ultrathin nanosheets/CFP	800	1 M KOH	10	390	[11]
$NiCoO_2$	1.03	1 M NaOH	10	390	[12]
Porous β-$Ni(OH)_2$ nanosheets	0.22	1 M KOH	10	415	[13]
Co-N/C 800	0.24	0.1 M KOH	10	510	[14]

References

1. Fei, H.; Dong, J.; Arellano-Jiménez, M.J.; Ye, G.; Kim, N.D.; Samuel, E.L.; Peng, Z.; Zhu, Z.; Qin, F.; Bao, J.; et al. Atomic cobalt on nitrogen-doped graphene for hydrogen generation. *Nat. Commun.* **2015**, *6*, 8668.
2. Xi, W.; Ren, Z.; Kong, L.; Wu, J.; Du, S.; Zhu, J.; Xue, Y.; Meng, H.; Fu, H. Dual-valence nickel nanosheets covered with thin carbon as bifunctional electrocatalysts for full water splitting. *J. Mater. Chem. A* **2016**, *4*, 7297–7304.
3. Xu, Y.; Tu, W.; Zhang, B.; Yin, S.; Huang, Y.; Kraft, M.; Xu, R. Nickel Nanoparticles Encapsulated in Few-Layer Nitrogen-Doped Graphene Derived from Metal–Organic Frameworks as Efficient Bifunctional Electrocatalysts for Overall Water Splitting. *Adv. Mater.* **2017**, *29*, 1605957.
4. Ai, L.; Tian, T.; Jiang, J. Ultrathin graphene layers encapsulating nickel nanoparticles derived metal-organic frameworks for highly efficient electrocatalytic hydrogen and oxygen evolution reactions. *ACS Sustain. Chem. Eng.* **2017**, *5*, 4771–4777.
5. Wang, J.; Xie, Y.; Yao, Y.; Huang, X.; Willinger, M.; Shao, L. Ni/NiO nanoparticles on a phosphorous oxide/graphene hybrid for efficient electrocatalytic water splitting. *J. Mater. Chem. A* **2017**, *5*, 14758–14762.
6. Faisal, S.N.; Haque, E.; Noorbehesht, N.; Liu, H.; Islam, M.M.; Shabnam, L.; Roy, A.K.; Pourazadi, E.; Islam, M.S.; Harris, A.T.; Minett, A.I. A quadrafunctional electrocatalyst of nickel/nickel oxide embedded N-graphene for oxygen reduction, oxygen evolution, hydrogen evolution and hydrogen peroxide oxidation reactions. *Sustain. Energy Fuels* **2018**, *2*, 2081–2089.
7. Oluigbo, C.J.; Xie, M.; Ullah, N.; Yang, S.; Zhao, W.; Zhang, M.; Lv, X.; Xu, Y.; Xie, J. Novel one-step synthesis of nickel encapsulated carbon nanotubes as efficient electrocatalyst for hydrogen evolution reaction. *Int. J. Hydrogen Energ.* **2019**, *44*, 2685–2693.
8. Fei, H.; Yang, Y.; Peng, Z.; Ruan, G.; Zhong, Q.; Li, L.; Samuel, E.L.G.; Tour, J.M. Cobalt nanoparticles embedded in nitrogen-doped carbon for the hydrogen evolution reaction. *ACS Appl. Mater. Interfaces* **2015**, *7*, 8083–8087.
9. Wang, Y.; Wang, Z.; Yang, Q.; Hua, A.; Ma, S.; Zhang, Z.; Dong, M. Edge-oriented MoS_2 supported on nickel/carbon core-shell nanospheres for enhanced hydrogen evolution reaction performance. *New J. Chem.* **2019**, *43*, 6146–6152.
10. Qian, Y.; Huang, S.; Li, D. Preparation of Nickel-Decorated and Nitrogen-Doped Carbon Nanotubes for Highly Efficient Hydrogen Evolution Reaction by Hydrothermal Method. *Energy Fuels* **2020**, *34*, 10241–10246.
11. Ye, Z.; Li, T.; Ma, G.; Dong, Y.; Zhou, X. Metal-Ion (Fe, V, Co, and Ni)-Doped MnO_2 Ultrathin Nanosheets Supported on Carbon Fiber Paper for the Oxygen Evolution Reaction. *Adv. Funct. Mater.* **2017**, *27*, 1704083.
12. Jung, S.; McCrory, C.C.L.; Ferrer, I.M.; Peters, J.C.; Jaramillo, T.F. Benchmarking nanoparticulate metal oxide electrocatalysts for the alkaline water oxidation reaction. *J. Mater. Chem. A* **2016**, *4*, 3068–3076.
13. Liang, H.; Li, L.; Meng, F.; Dang, L.; Zhuo, J.; Forticaux, A.; Wang, Z.; Jin, S. Porous two-dimensional nanosheets converted from layered double hydroxides and their applications in electrocatalytic water splitting. *Chem. Mater.* **2015**, *27*, 5702–5711.
14. Hu, W.; Wang, Q.; Wu, S.; Huang, Y. Facile one-pot synthesis of a nitrogen-doped mesoporous carbon architecture with cobalt oxides encapsulated in graphitic layers as a robust bicatalyst for oxygen reduction and evolution reactions. *J. Mater. Chem. A* **2016**, *4*, 16920–16927.
